# Organizational change: challenges for workplace psychosocial risks and employee mental health

**DOI:** 10.1186/s12889-024-19815-w

**Published:** 2024-09-11

**Authors:** Insa Backhaus, Andrea Lohmann-Haislah, Hermann Burr, Karina Nielsen, Cristina di Tecco, Nico Dragano

**Affiliations:** 1 Centre for Global Health Inequalities Research, Department of Sociology and Political Science, Norwegian University of Science and Technology, Trondheim, Norway; 2 Institute of Medical Sociology, Centre for Health and Society, Medical Faculty and University Hospital, Heinrich Heine University Düsseldorf, Düsseldorf, Germany; 3 Federal Institute for Occupational Safety and Health, Unit Psychosocial Factors and Mental Health, Berlin, Germany; 4 Institute of Work Psychology, Sheffield University Management School, University of Sheffield, Sheffield, UK; 5 Italian Workers’ Compensation Authority (INAIL), Department of Occupational and Environmental Medicine, Epidemiology and Hygiene, Rome, Italy

**Keywords:** Organizational change, Psychosocial risks, Changes at the workplace, Mental health

## Abstract

**Background:**

Constant organizational change is the norm in many companies today. At present, evidence on the impact of organizational change on psychosocial risks at work and employee mental health is limited. We investigate organizational change and its association with psychosocial risks and mental health in three consecutive surveys covering 12 years.

**Methods:**

The study was based on data from three cross-sectional waves (2006, 2012, 2018) of the German BIBB/BAuA Employment Survey, comprising 53,295 employees. Four change indicators (i.e., introduction of new software, changes in goods and services produced/provided, downsizing and restructuring), five indicators of psychosocial risks (i.e., time pressure, interruptions, multitasking, working to the limits of capability, and working very quickly) and four mental health indicators (i.e., sleep disturbances, nervousness, tiredness and depressive symptoms) were investigated. We applied Poisson regression analysis to examine associations between organizational change, psychosocial risks, and mental health.

**Results:**

According to the pooled analysis of all three waves, the majority of employees reported having experienced at least one organizational change, such as downsizing or restructuring, between 2006 and 2018. Organizational change was negatively associated with psychosocial risks (e.g., working to the limits of one’s capability, PR: 1.66; 95% CI: 1.48–1.86) and with employee mental health (PR: 1.82; 95% CI: 1.61–2.04).

**Conclusions:**

Organizational change is omnipresent in the modern economy. Our research suggests that transformation processes in organizations can bear risks to employees’ health as psychosocial risks increase. Therefore, companies planning organizational change should accompany such processes with occupational health and safety measures.

## Abstract (significance)

**What is already known on this topic**

• Globalization, constant technological developments and the volatile economic and geopolitical climate require constant organizational change.

• The implications of organizational changes on psychosocial risks and employee wellbeing are not well known.

**What this study adds**

• This study showed that organizational change is associated with adverse psychosocial risks and poor mental health.

• Over the 12-year period investigated in this study, the gap in poor mental health between employees experiencing change and those not experiencing change did not increase.

**How this study might affect research, practice or policy**

• The findings of our study suggest that organizational change is associated with both adverse psychosocial risks at work and poor employee mental health.

• Therefore, the implementation of organizational change must be closely followed by occupational health and safety services to prevent an increase in psychosocial risks as well as to prevent a decline of employee mental health.

## Introduction

Organizational change is ubiquitous and far-reaching in the modern economy. Globalization, constant technological progress as well as a volatile economic and geopolitical climate have fundamentally changed the working world over the past several decades, but only a few studies have investigated the relationship between organizational change and psychosocial risks at work and employee mental health.

Organizational change refers to the process of companies or organizations changing their structures, strategies, procedures or cultures through measures such as downsizing, restructuring, outsourcing and mergers [1]. While it can have positive outcomes for the company, such as improved efficiency, performance and profitability, the international literature suggests that organizational change is often implemented at the cost of employees’ working conditions and health [2–4].

In terms of working conditions, it is well-acknowledged that in industrialised countries, risks have changed from those predominantly including physical risks (i.e., physical working conditions) to those including psychosocial risks (i.e., psychosocial working conditions) such as time pressure, multitasking and work intensity, over the past decades [5]. However, findings regarding trends and developments in psychosocial risks are often ambiguous, suggesting both an improvement and a worsening of psychosocial risks at work. As such a some studies point towards decreasing or stable trends [6, 7], while other studies suggest a deterioration and an increase psychosocial risks such as job strain, working intensity and working hours [8–10].This has, among other causes, been attributed to organizational change, digitalization and general changes in the labour market [4, 9, 11]. Research has linked organizational change to role unclarity, job insecurity, intensification of time pressure and job strain, and reduced social support and job control [12]. In an early study, Kivimaeki et al. (2001), for instance, found that downsizing significantly predicted job insecurity and job control [12]. This is important because psychosocial risks at work have been acknowledged as an important determinant of health and a multitude of studies has linked unfavourable psychosocial working conditions (e.g., high job demand and low control) with health conditions such as hypertension, cardiovascular disease, obesity and musculoskeletal disorders (e.g., lower back pain and upper limb pain) [13–18]. In addition to affecting physical health, poor psychosocial working conditions have also been closely linked to mental health problems for which an increase has been detected across Europe [46].

Researchers in the fields of occupational and public health have followed the rising trend of mental health problems, suggesting that changes in working conditions may offer a potential explanation. Like many other European countries, the German labour market has undergone significant changes over the past few decades. These changes were primarily influenced by technological advancements, economic changes, policy reforms and lately the COVID-19 pandemic. Particularly notable were policy reforms enacted between 2002 and 2004, known as the ‘Hartz reforms’. These reforms were implemented to cut unemployment benefits and to allow for greater labour market flexibility, but also resulted in an increased use of flexible forms of employment, such as temporary and part-time contracts [19]. Researchers have also argued that because of these reforms job insecurity increased, which in turn led to more demand pressure and competition as employees faced constant pressure to keep their positions - which in turn is put forward to have increased job strain [19].

Despite an increasing interest in the effects of organizational change on psychosocial risks at work and employees’ health, studies investigating trends of organizational change and their effect on psychosocial risks and health remain scarce [20, 21]. Furthermore, available research often investigates either the association between organizational change and health or the association between psychosocial working conditions and health but does not simultaneously look at the impact of organizational change on psychosocial risks and health. However, since employees are increasingly confronted with organizational change and the upheavals of restructuring, downsizing and mergers [21], solid knowledge about the trends of organizational change and its association with psychosocial risks at work and health is needed to maintain a healthy work environment and to implement interventions that can buffer the potential adverse effects of organizational change.

We, therefore, also explore whether different forms of changes in the work environment between 2006 and 2018 are linked to psychosocial risks at work and employee mental health. To do so, we first provide a description of trends of organizational changes and psychosocial risks at work, followed by an analysis investigating the association between organizational changes and psychosocial risks at work, and an examination of the link between changes and employee mental health. We examine various organizational change events (i.e., downsizing, outsourcing, continuous improvement, and process reengineering), psychosocial risks (i.e., tight deadline or performance pressure, work interruptions, multitasking, working very quickly and working to the limits of capability) and mental health among a German sample of employees.

## Methods

### Data

For the present study, we used data from three waves (2006, 2012, 2018) of the German BIBB/BAuA Employment Survey [22–24]. Originally launched in 1979, this survey has been conducted regularly (approximately every five years) since 2006 in cooperation between the Federal Institute for Vocational Education and Training (BIBB) and the Federal Institute for Occupational Safety and Health (BAuA) as the BIBB/BAuA Survey of Employed Persons. The survey focuses on questions relating to the workplace (e.g., work activities, workload factors and resources), health impairments, as well as occupational qualifications and developments. The survey provides a representative sample of the working population in Germany.

The target subjects of the sample were drawn in 2006 and 2011 using the Gabler-Häder method; [25] in 2018, a further development of the Gabler-Häder method of the ADM [26] was used in combination with the Kish method [27]. In each case, around 20,000 employed persons aged 15 and over with a working time of at least ten hours per week were surveyed (including family members helping, excluding volunteers or employees in their first training). Non-German citizens were only included in the survey if they had sufficient knowledge of German. However, only employees are included in the following analyses and self-employed persons, freelancers and family workers are not taken into account. This results in the following sample sizes: n (2006) = 17,612; n (2012) = 17,799; n (2018) = 17,884.

Computer Assisted Telephone Interview (CATI) was used as survey method. In 2018, the telephone survey was conducted for the first time using a dual-frame sample (i.e., recruitment and interviewing using landlines and mobile phone numbers). On average, an interview took approximately 40 min to complete.

### Measures

#### Organizational change

Previous studies have suggested that a correlation between change characteristics (e.g., downsizing) and mental health as well as psychosocial risks exists. To investigate the link between organizational change and health as well as psychosocial risks at work, we used four items assessing changes in the workplace in the last two years with a binary response option (i.e., yes, no). These changes include (a) the introduction of new computer programs, (b) changed services, (c) downsizing and (d) restructuring. Furthermore, since we were particularly interested in the cumulative impact of multiple organizational changes on mental health and psychosocial risks, and in how an environment of change is associated with mental health and psychosocial risks at work, we summed these four items as a score (range: 0–4; KR-20 = 0.5 ) with the title “organizational change” and then dichotomized the score so that 0 represents ‘no organizational change’ and that the values 1–4 represent ‘at least one organizational change’.

#### Psychosocial risks

Psychosocial risks at work refer to psychosocial factors that can cause harm to the individual worker or the entire organization. In the present study, we focused on five indicators of individual psychosocial risks at the workplace that have been suggested to follow organizational change and that have been considered particularly relevant in the German work environment [2, 12, 28–30]. These indicators were derived from survey questions inquiring whether the individual had experienced (a) strong deadline or performance pressure, (b) work interruptions, (c) multitasking, (d) having to work quickly and (e) working to the limits of capability. All items were measured on a four-point Likert scale with the response options ‘often’, ‘sometimes’, ‘seldom’, and ‘never’. In line with previous works of the Federal Institute for Occupational Safety and Health (BAuA) [30], we dichotomized these single items for the analyses with 0 representing ‘sometimes, seldom and never’ and 1 representing ‘often’.

#### Mental health

Mental health information was derived from a survey question asking about health complaints that had occurred in the last twelve months either at work or on workdays by answering options “yes” or “no”. This list contained four mental health indicators that were available in all waves of the survey. These complaints include (a) sleep disturbances, (b) nervousness or irritability, (c) general tiredness, faintness or exhaustion and (d) depressiveness. The answer options were binary with ‘no’ (0) and ‘yes’ (1). For the analyses, we created a score (range: 0–4, Cronbach’s alpha = 0.73) by summing these items and then dichotomized the score so that the values 0–2 indicate ‘good mental health’ whereas the scores 3–4 imply ‘poor mental health’.

#### Covariates

For the analyses we considered the following covariates (a) gender (male, female), (b) age (four age groups in years: 15–29, 30–49, 50–65, > 65), (c) economic sector (4 sectors = public service, industry, skilled trades, service sector and other sectors and d) professional qualification based on the 2010 German Classification of Occupations (i.e., KldB 2010). The KldB differentiates between four requirement levels: level (1) unskilled, low-complexity routine tasks, (2) skilled, more technical tasks requiring at least two or three years of vocational training, (3) complex tasks involving special knowledge, which requires at least master craftsman or technician training and (4) highly complex tasks requiring at least a higher education degree.

### Statistical analysis

We performed descriptive analyses to describe the sample and to explore the prevalence of organizational change, psychosocial risks, and mental health. We then regressed organizational change and psychosocial risks by applying Poisson regressions. We used Poisson regressions for binary outcomes because they allow the estimation of prevalence ratios (PRs) and are easier to interpret than odds ratios [31]. We adjusted the Poisson regression models for age (15–29, 30–49, 50–65 and > 65 years), professional qualification (level 1: unskilled, low-complexity routine tasks, level 2: skilled, more technical tasks requiring at least two or three years of vocational training, level 3: complex tasks involving special knowledge, which require at least master craftsman or technician training and level 4: highly complex tasks requiring at least a higher education degree), employment sector (Trade/Commerce, Civil Services, Industry, Services, Other) and survey wave (in case of pooled analyses). We also computed predicted probabilities for mental health and organizational change. The changes in the predicted probabilities between the waves are expressed by average marginal effects (AMEs) to facilitate interpretation. Moreover, to simplify the interpretation of the results we used binary outcomes for all analyses. Regarding the descriptive analyses, the percentages reported below are weighted values based on unweighted n’s; inferential statistical analyses are always based on unweighted values. All analyses were performed using STATA version 16.0 (StataCorp LP, College Station, TX).

To account for sampling bias, all surveys used a multistage, iterative weighting method. That is, an iterative weighting process is set up, the result of which are weighting factors that adjust the realized sample to all specified target distributions with predefined precision and minimum variance. The characteristic distributions of the variables age, federal state, German/non-German, marital status, gender, highest school-leaving qualification, and position in occupation (each from the micro censuses of the Federal Statistical Office: 2005 for the 2006 survey, 2011 for the 2012 survey, and 2017 for the 2018 survey) served as reference data.

## Results

### Sample characteristics

Table 1 provides a description of the sample. The combined sample (*n* = 53,295) included more men (54.2%) than women (45.8%). Most of the employees were between the ages 30–49 years (52.7%) (mean age: 44.4 years, SD: 11.02), and one-fourth of the employees worked either in the civil service sector (25%) or the industrial sector (26%). A total of 10,850 (20.1%) employees reported poor mental health.

**Table 1:** Sample characteristics (*n* = 53,295)

	Total		2006 (*n* = 17,612)		2012 (*n* = 17,799)		2018 (*n* = 17,884)	
	No.	%^*^	No.	%^*^	No.	%^*^	No.	%^*^
**Gender**								
*Male*	25,815	54.2	8,832	55.2	8,214	53.9	8,769	53.5
*Female*	27,480	45.8	8,780	44.8	9,585	46.1	9,115	46.5
**Age categories** ^a^								
*15–29 years*	5,913	16.6	2,797	16.8	1,582	17.0	1,534	16.0
*30–49 years*	27,589	52.7	10,863	58.3	8,987	52.8	7,739	47.0
*50–65 years*	19,246	30.3	3,915	24.7	6,996	29.8	8,335	36.3
*> 65 years*	269	0.5	37.0	0.3	108	0.5	124	0.7
**Classification of occupations** ^b^								
*Unskilled*,* low-complexity routine tasks*	3,688	9.1	1,351	9.1	1,241	8.9	1,096	9.2
*Skilled*,* more technical tasks requiring at least two or three years of vocational training*	29,231	61.6	9,606	58.6	9,826	58.0	9,799	69.4
*Complex tasks involving special knowledge*,* which require at least master craftsman or technician training;*	7,140	12.4	2,855	14.4	2,974	14.9	1,311	7.2
*Highly complex tasks requiring at least a higher education degree*	9,808	15.7	3,741	17.5	3,572	17.2	2,495	11.6
*No classification*	616	1.3	59	0.4	186	1.0	371	2.6
**Economic sector**								
*Civil service*	15,759	26.0	5,112	26.4	5,180	25.7	5,467	25.8
*Industry*	11,376	25.0	4,156	25.2	3,817	27.6	3,403	22.3
*Trade/Commerce*	9,926	22.7	3,669	23.5	3,417	23.3	2840	21.4
*Services*	12,662	23.1	4,362	23.1	3,906	21.3	4,394	24.8
*Other*	1,551	3.2	291	1.9	342	2.1	918	5.6
**Psychosocial risks**								
**Strong deadline or performance pressure** ^c^								
*Never/seldom/sometimes*	24,850	49.1	7,821	46.5	8,284	48.4	8,745	52.3
*Often*	28,427	50.9	9,789	53.5	9,508	51.6	9,130	47.7
**Disturbances/Interruptions** ^d^								
*Never/seldom/sometimes*	27,060	54.1	8,708	52.7	9,402	55.8	8,950	53.8
*Often*	26,206	45.9	8,901	47.3	8,387	44.2	8,918	46.2
**Multitasking** ^e^								
*Never/seldom/sometimes*	19,510	40.8	6,636	41.2	6,774	41.5	6,100	39.6
*Often*	33,757	59.2	10,971	58.8	11,013	58.5	11,773	60.4
**Working to the limits of capability** ^f^								
*Never/seldom/sometimes*	44,279	83.7	14,695	83.4	14,712	83.7	14,872	83.9
*Often*	8,970	16.3	2,909	16.6	3,070	16.3	2,991	16.1
**Working very quickly** ^g^								
*Never/seldom/sometimes*	32,602	60.9	9,791	55.5	10,874	61.1	11,937	66.1
*Often*	20,574	39.1	7,797	44.5	6,877	38.9	5,900	33.9
**Organizational change**								
**Organizational change (score** ^**^ **)**								
*No changes*	11,439	22.5	3,012	19.1	4,085	25.8	4,342	27.7
*At least one change*	39,498	77.5	13,811	80.9	12,931	74.2	12,756	72.3
**Introduction of new computer programmes** ^h^								
*No*	26,102	53.5	8,071	49.7	9,126	55.5	8,905	55.2
*Yes*	25,716	46.5	8,979	50.3	8,172	44.5	8,565	44.8
**Changed services** ^i^								
*No*	37,375	72.7	11,918	69.5	12,703	73.8	12,754	74.8
*Yes*	15,268	27.3	5,518	30.5	4,875	26.2	4,875	25.2
**Downsizing** ^j^								
*No*	28,710	56.2	8,962	53.3	9,869	57.3	9,879	58.0
*Yes*	24,318	43.8	8,577	46.7	7,842	42.7	7,899	42.0
**Restructuring** ^k^								
*No*	34,525	65.3	9,725	55.2	11,856	67.8	12,944	73.0
*Yes*	18,322	34.7	7,775	44.8	5,807	32.2	4,740	27.0
**Mental health**								
*Good mental health*	42,237	79.9	14,566	83.2	13,761	78.7	13,910	77.8
*Poor mental health*	10,850	20.1	3,014	16.8	3,965	21.3	3,871	22.2

*Note*: cases missing a) n = 278 (0.6%); b) n = 2,812 (5.3%); c) n = 18 (0.03%); d) n = 29 (0.05%); e) n = 28 (0.05%); f) n = 46 (0.1%); g) n = 119 (0.2%); h) n = 1477 (3.6%); i) n = 652 (1.6%); j) n = 267 (0.7%); k) n = 448 (1.0%); l) n = 114 (0.3%); m) n = 105 (0.3%); n) n = 104 (0.2%); o) n = 104 (0.2%); * Percent are weighted; ** Summary score of the four items on change, dichotomized 0–2 changes = low, 3–4 = high

### Trends in organizational change, psychosocial risks and mental health

Employees most frequently reported the introduction of new computer programs (46.5%), followed by downsizing (43.8%) and restructuring (34.7%). Looking at trends of organizational change across the survey, we can observe a slight decrease since 2006. As such, the percentage of employees experiencing changes at the workplace decreased by 9% points from 2006 (80.9%) to 2018 (72.3%).Of the psychosocial risks considered, 59.2% of employees reported working often on several tasks simultaneously (i.e., multitasking), 50.9% reported often strong deadlines or performance pressure, 45.9% reported frequent interruptions in their work, 16.3% reported working often to the limits of capability and 39.1% said they often had to work very quickly (Table 1). Looking at the development of psychosocial risks over all three survey waves, we can note a slight decrease in the proportions of those affected often by strong deadlines, working to the limits of capability and working very quickly. A nonlinear trend can be noted for multitasking and disturbances and interruptions. Among all, the most significant decrease between 2006 and 2018 can be noted in the requirement ‘working very quickly’ (-10.6% points), followed by ‘strong deadline or performance pressures’ (-5.8% points) (Table 1).

### Association between organizational change and psychosocial risks

Table 2 shows the results of the Poisson regression examining the associations between organizational change as well as the organizational change indicators with individual psychosocial risks. All organizational change indicators were significantly associated with the psychosocial risks investigated. For example, downsizing was significantly associated with working to the limits of capability (PR: 1.55; 95% CI: 1.44–1.67). Experiencing a number of organizational changes was associated with increased prevalence ratios of all investigated workplace psychosocial risks independent of the survey wave (Table 2). For example, the PR for the association between experiencing change at the workplace and being often disturbed at work was 1.59 (95% CI: 1.49–1.70) and 1.66 (95% CI: 1.48–1.86) for working often to the limits of one’s capability.

**Table 2:** Results of the Poisson regressions showing prevalence ratios (PR) and 95% confidence intervals (CI) for the association between organizational change(s) and single psychosocial risks at work (often)

	Strong deadline or performance pressure		Disturbances/ Interruptions		Multitasking		Working to the limits of capability		Working very quickly	
	PR	95% CI	PR	95% CI	PR	95% CI	PR	95% CI	PR	95% CI
**Organizational change** ^**a**^										
*No changes (ref)*	1.00		1.00		1.00		1.00		1.00	
*At least one change*	1.39	[1.31–1.47]	1.59	[1.49–1.70]	1.26	[1.19–1.33]	1.66	[1.48–1.86]	1.37	[1.28–1.46]
**Introduction of new computer programs**										
*No (ref)*	1.00		1.00		1.00		1.00		1.00	
*Yes*	1.18	[1.14–1.23]	1.30	[1.24–1.36]	1.15	[1.11–1.20]	1.11	[1.03–1.20]	1.17	[1.11–1.22]
**Changed services**										
*No (ref)*	1.00		1.00		1.00		1.00		1.00	
*Yes*	1.22	[1.17–1.27]	1.25	[1.20–1.31]	1.18	[1.13–1.23]	1.40	[1.30–1.51]	1.23	[1.17–1.29]
***Restructuring***										
*No (ref)*	1.00		1.00		1.00		1.00		1.00	
*Yes*	1.19	[1.14–1.24]	1.24	[1.19–1.29]	1.08	[1.04–1.12]	1.42	[1.32–1.52]	1.24	[1.18–1.29]
**Downsizing**										
*No (ref)*	1.00		1.00		1.00		1.00		1.00	
*Yes*	1.27	[1.22–1.32]	1.35	[1.29–1.40]	1.21	[1.17–1.26]	1.55	[1.44–1.67]	1.25	[1.19–1.30]

*Note*: Computation based on separate Poisson regressions adjusted for age, gender, classification of occupation and survey year; a) based on a summary index of four organizational change indicators (i.e., the introduction of new computer programs, changes in goods and services, downsizing and restructuring) and then dichotomized

### Association between organizational change and mental health

Table 3 shows the results of the Poisson regression analyses examining the associations between changes at the workplace and employee mental health. Experiencing change at the workplace was significantly associated with poor mental health (PR: 1.82, 95% CI: 1.61–2.04). Specifically, experiencing organizational change was associated with sleep disturbances (PR: 1.68; 95% CI: 1.51–1.86), nervousness (PR: 1.56; 95% CI: 1.45–1.70), tiredness (PR: 1.30; 95% CI: 1.22–1.39) and depressiveness (PR: 1.53; 95% CI: 1.38–1.71).

**Table 3:** Results of the Poisson regressions showing prevalence ratios (PR) and 95% confidence intervals (CI) for the association between organizational change and employee mental health

	Poor Mental health^b^		Sleep disturbances		Nervousness		Tiredness		Depressiveness	
	PR	95% CI	PR	95% CI	PR	95% CI	PR	95% CI	PR	95% CI
**Organizational change** ^**a**^										
*No changes (ref)*	1.00		1.00		1.00		1.00		1.00	
*At least one change*	1.82	[1.61–2.04]	1.67	[1.50–1.85]	1.56	[1.43–1.70]	1.31	[1.23–1.40]	1.54	[1.38–1.71]
**Wave**										
*2006 (ref)*	1.00		1.00		1.00		1.00		1.00	
*2012*	1.38	[1.20–1.59]	1.41	[1.25–1.60]	1.05	[0.94–1.17]	1.07	[0.99–1.16]	1.25	[1.10–1.42]
*2018*	1.51	[1.32–1.74]	1.65	[1.46–1.86]	1.09	[0.98–1.22]	1.18	[1.09–1.28]	1.18	[1.03–1.34]
**Economic sector**										
*Trade/Commerce (ref)*	1.00		1.00		1.00		1.00		1.00	
*Civil services*	1.10	[1.03–1.17]	1.27	[1.20–1.34]	1.05	[1.00–1.11]	1.09	[1.05–1.14]	0.97	[0.92–1.03]
*Industry*	1.01	[0.94–1.08]	1.15	[1.08–1.22]	0.98	[0.93–1.04]	1.00	[0.95–1.04]	0.95	[0.90–1.02]
*Services*	1.03	[0.96–1.09]	1.15	[1.08–1.22]	0.99	[0.94–1.04]	1.02	[0.98–1.07]	0.92	[0.86–0.98]
*Other*	1.04	[0.92–1.19]	1.22	[1.09–1.36]	0.98	[0.88–1.10]	1.00	[0.92–1.09]	0.97	[0.86–1.11]
Observations	46,251		46,176		46,191		46,189		46,190	

*Note:* Computation based on Poisson regressions, adjusted for age, gender, classification of occupation and survey year; (a) based on a summary index of four organizational change indicators (i.e., introduction of new computer programs, changes in goods and services, downsizing, and restructuring) and then dichotomized; (b) based on a summary index of four mental health indicators (i.e., sleep disturbances, nervousness/irritability, tiredness and depressiveness) and then dichotomized

**Fig. 1:**
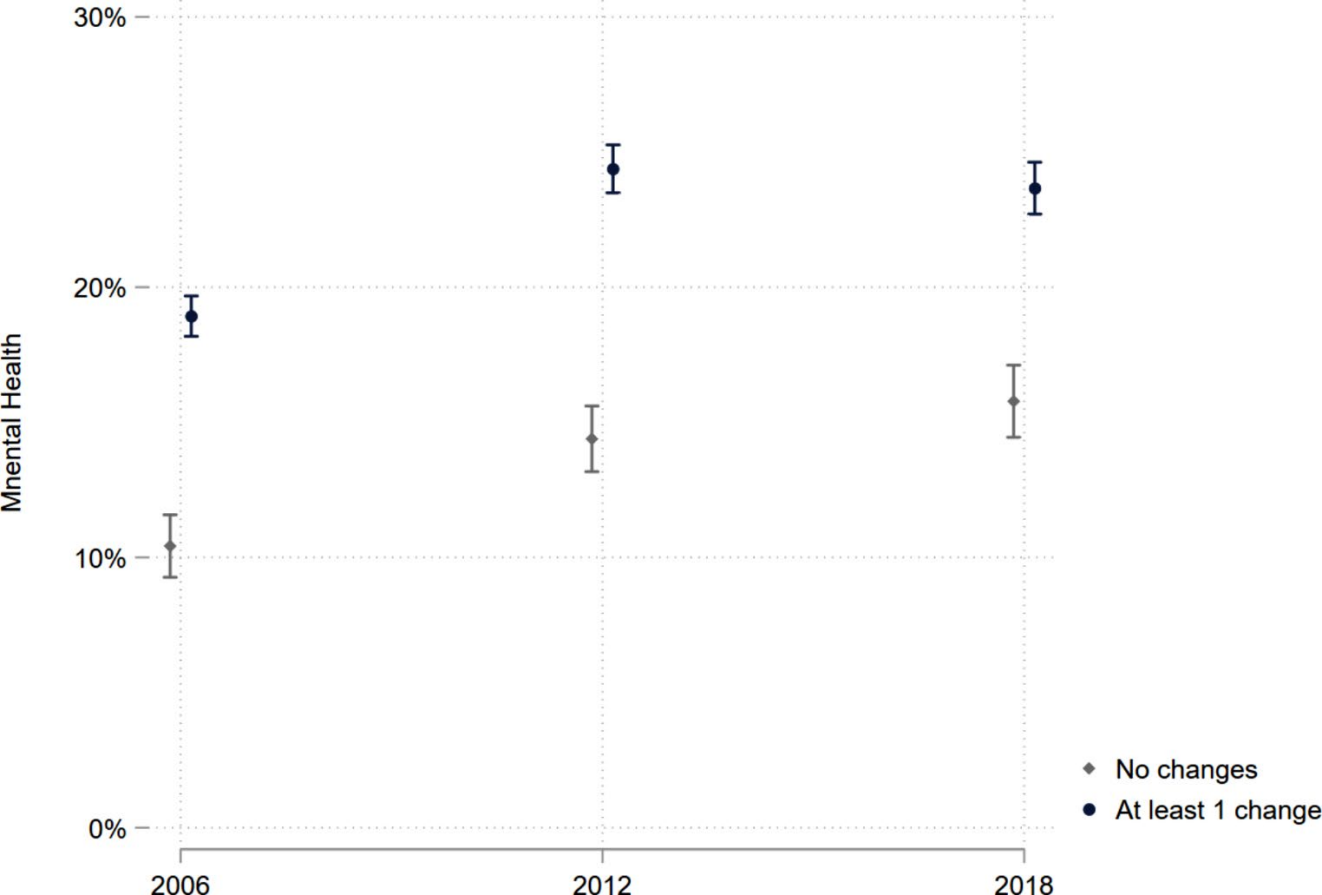
Predicted probabilities of poor mental health by organizational change Legend: Predicted probabilities and their 95% confidence intervals. Computation based on Poisson regressions analysis as specified in Table3, adjusted for age, classification of occupation and employment sector. Error bars represent 95% confidence intervals

Table 4 and Fig. 1 show that poor mental health increased from 2006 to 2018 for all employees – with a particular increase between 2006 and 2012. However, the pattern is not uniformly distributed within each wave and between groups. First, in each wave, the predicted probabilities of poor mental health were higher among employees experiencing organizational change than those not experiencing change. To exemplify, in 2012, the predicted probability for poor mental health among employees experiencing organizational change was 24.4 (95% CI: 23.5–25.3), whereas it was 14.4 (95% CI: 13.2–15.6) for those not experiencing change, corresponding to a difference of 10% (*p* < 0.001). Second, the results suggest that the predicted probabilities for poor mental health rose specifically in the year 2012, however, declining in 2018 for employees experiencing at least one change, but increasing further for employees experiencing no change. Third, unlike expected, we could not find a significant difference in the increase in poor mental health between employees experiencing change and those not experiencing change over time. In other terms, the gap in poor mental health between employees experiencing change and those not experiencing change did not increase between 2006 and 2018. This pattern is illustrated in the bottom right-hand corner of Table 4, which shows the value of the difference in trends (from 2006 to 2018) between those experiencing change and those not experiencing change.

**Table 4:** Predicted probabilities of poor mental health (summary score, dichotomized) by organizational change (*n* = 50,927)

	2006 Predicted Probability (95% CI)	2012 Predicted Probability (95% CI)	2018 Predicted Probability (95% CI)	AME 2018 vs. 2006 (*p*-value)	AME 2012 vs. 2006 (*p*-value)
Change at the workplace					
*No changes*	10.4 (9.3–11.6)	14.4 (13.2–15.6)	15.8 (14.4–17.1)	5.4 (*p* < 0.001)	3.9 (*p* < 0.001)
*At least one change*	18.9 (18.2–19.7)	24.4 (23.5–25.3)	23.7 (22.7–24.6)	4.8 (*p* < 0.001)	5.5 (*p* < 0.001)
*AME*	8.5 (*p* < 0.001)	10.0 (*p* < 0.001)	7.9 (*p* < 0.001)	-0.6 (*p* = 5.567)	1.5 (*p* = 0.151)

*Note:* Estimates are based on Poisson regressions on the association between covariates and psychosocial risks, adjusted for age, gender, classification of occupation and employment sector, 95% CI = 95% Confidence Interval, AME = Average Marginal Effects

## Discussion

Global developments have led to organizational changes affecting the way work is performed. In the present study, we examined trends in organizational change and psychosocial risks as well as the associations between organizational change and psychosocial risks and employee’s mental health. While many studies focus on the development of adverse working conditions, less evidence is available on the link between organizational change and psychosocial risks at work as well as on the link between organizational change and employees’ mental health. Our study offers two important findings. First, we showed that organizational change is negatively associated with psychosocial risks at work and second, we discovered that organizational change is associated with poorer employee mental health. Against our expectation, the extend of organizational change remained the same between 2006 and 2018 in Germany.

With regard to psychosocial risks, our results are in line with previous studies, linking organizational change to increases in psychosocial risks at work [3]. In a longitudinal analysis, Conway et al., for instance, found that organizational restructuring increased the risk of workplace bullying [32]. Fløvik and colleagues (2019) found that job demands increased following the implementation of various types of organizational change (e.g., restructuring and downsizing) [2]. The authors argue that this increase might be due to the introduction of new change-related tasks, which come on top of the usual tasks during the process of organizational change. This could also explain our finding that multitasking and working to the limits of capability increased as part of organizational change. Apart from that, other explanations may be that employment relations change during the process of organizational change [33, 34]. Moreover, change may come with uncertainty, which can trigger feelings of stress especially when it threatens the existence of one’s job and loss of income [4]. The uncertainty surrounding organizational change may have a negative impact on employees. We found that employees experiencing organizational change report poorer mental health than employees not experiencing change. There are several mechanisms through which organizational change may affect employee mental health [12, 35–37]. Restructuring, for example, can increase workload, which may exacerbate perceived stress. Downsizing can cause job insecurity, which is linked to poorer mental and self-rated health [12, 35, 38]. A longitudinal study from Finland, for instance, found a decline in self-rated health among employees who had experienced downsizing. The authors linked this decline to changes in psychosocial work factors such as job insecurity and a reduction in job control [12].

It is important to recognize that the impact of organizational change might differ depending on the type of change implemented and the context (e.g., employment sector) in which it occurs. As such the introduction of new technology can have different implications in industrial production compared to the civil service sector. While, for example, in the manufacturing industry, the introduction of new technology may involve the implementation of automation technology that could lead to concerns about layoffs, in the civil service sector, the introduction of new technologies might relate to the implementation of new computer programs that could cause fear among employees of being unskilled as well as technostress (i.e., the stress and negative psychological effects associated with the use of technology). Therefore, both the nature of the organizational change, its unique characteristics as well as the context in which it takes place may play a crucial in determining the overall impact on employees. In the present study, we were unable to investigate the unique differences within organizational change and it was out of scope to examine differences by employment sector or occupation. Still, a deeper understanding of how the impact of organizational might differ is needed.

An unexpected and interesting finding is that neither organizational change nor psychosocial risks increased in Germany over the twelve years under study; however, they remained stable at a high level and in some cases decreased. Specifically, “working very quickly” decreased by nearly 11% points from 2006 to 2018, while multitasking remained high but stable, whereas, mounting evidence from Europe shows that psychosocial risks have increased over the past 25 years [9–11, 39, 40]. More specifically, Lopes and colleagues demonstrated by analysing data from several European countries that work autonomy has declined and work pressure has increased in most EU countries since 1995 [9]. Similarly, Rigó et al. [10]. found that work pressure and work stress increased over the period 1995 to 2015 and Myers et al. found that from 2002 to 2014, job strain, low job control and work-family conflict increased [40]. Regarding organizational change, trend studies are, to the best of our knowledge, lacking. There is increasing concern about the quickly changing working world and its possible consequences for employees. In the present study, the amount of organizational change remained stable, and we can only speculate about the underlying reasons. First, it is possible that the Bibb/BAuA survey does not fully capture organizational change. For instance, while it investigates the introduction of new technologies and computer programs, it may not represent the full spectrum of digitalization. Second, our data suggest that organizational change was most intense in 2006. It is likely that a great amount of organizational change took place in the period between 2008 and 2010 – the time of the economic crisis and recession – which was not captured in the Bibb/BAuA survey. During this period, many employees were affected by layoffs and restructuring processes. After the economic recession organizational change may have slowed down. Third, over the past few years Germany has experienced bureaucratization, which may have acted as a barrier and may have discouraged some companies from engaging in innovation and consequently introducing organizational change [41]. For instance, van Dijck and Steen (2023) put forward that strong bureaucracy can reduce organizational flexibility in an organization [41].

### Implications

In the present study, we were unable to investigate the underlying mechanisms between organizational change, psychosocial risks and health. To implement interventions targeted at reducing the potential adverse effects of organizational change, in-depth studies investigating “how” and “why” are needed. Nevertheless, our results suggest that organizational change is associated with both poor psychosocial risks and health. Therefore, changes in the working conditions necessitate a stronger assessment of occupational health and safety (OHS) and OHS services must adapt to these rapid changes [34]. At the same time, they should recognize new opportunities for effective interventions arising from new developments (e.g., app-based psychosocial risk assessments). For instance, the emerging use of information and communication technology can be accompanied by technology-induced stress (so-called ‘technostress’) [42] and an increasingly mobile workforce due to increasing possibilities for remote working, requires prevention strategies other than locally bound employees working at one identifiable workplace. OHS services must be aware of recent developments to meet the demands for prevention. Furthermore, the way in which organizational change is implemented can make a substantial difference as to whether the exposed employee will experience great levels of uncertainty or whether the organizational change is stressful. An employer plays a significant role in change processes and can help shape the working conditions of employees, and the adverse effects of organizational change, including uncertainty, might be reduced through timely, transparent and comprehensive communication [43, 44]. As such, studies examining the impact of restructuring and job insecurity suggest that individualized communication, including early and transparent communication about the changes, helped mitigate negative effects on employees who find themselves in restructuring processes [45].

## Strengths and limitations

Our study has several strengths and limitations that should be acknowledged when interpreting the findings. First, this study offers novel findings regarding specific indicators of organizational change and psychosocial risks as well as employee mental health in Germany. Despite the increasing interest in the effects of organizational change, trend studies on organizational change and psychosocial risks and mental health remain scarce. Second, the BIBB/BAuA employment survey is a representative survey that is unparalleled in terms of the number of participants and its differentiation in depicting the conditions in the world of work. Third, the regular implementation of the survey makes it possible to identify changes in the world of work. Nevertheless, there are some limitations to be considered. First, our results are country-specific and not generalizable to other European countries. The political and economic environment may affect the pace at which workplace changes are introduced and implemented. Second, the four items on organizational change are only a sample of changes and do not reflect possible complexities. Furthermore, the KR-20 reliability coefficient of the sum score was only low to moderate suggesting that the items used only have an average level of internal consistency and the score may not fully reflect employees’ experiences of change. This limitation should be noted when interpreting and drawing conclusions about the findings using the sum score.

Last, due to the cross-sectional data and the empirical approach chosen, the results presented do not allow any causal conclusions to be drawn. Thus, it cannot be ruled out that the estimated correlations may differ due to reverse causality or unobserved heterogeneity. In order to derive targeted implications, future studies using panel data or quasi-experimental methods should, therefore, concentrate on mapping long-term or permanent relationships and identifying causal effects. In addition, changes in the workplace would have to address the operationalization of digitalization and, in doing so, also pay attention to the most differentiated recording of the various technologies possible. However, the data are currently still insufficient and future studies should take these aspects into account.

## Conclusion

We found a negative association between frequent organizational change and psychosocial risks and employees’ mental health. This association was observed in three independent cross-sectional waves of a large population-based survey and was reinforced over time. To protect employees’ health during organizational change processes, it is therefore recommended that organizational processes are accompanied by occupational health and safety measures.

## Data Availability

The datasets generated and/or analyzed during the current study are available in the Data Archive for the Social Sciences (DAS) repository at GESIS.

## Abbreviations

CI: Confidence interval
PR: Prevalence ratio
AMEs: Average marginal effects
Ref: Reference category
OHS: Occupational health and safety
INAIL: Italian National Institute for Insurance against Accidents at Work
